# Molecular epidemiology and clinical characteristics of enteroviruses associated HFMD in Chengdu, China, 2013–2022

**DOI:** 10.1186/s12985-023-02169-x

**Published:** 2023-09-03

**Authors:** Qiuxia Yang, Fang Liu, Li Chang, Shuyu Lai, Jie Teng, Jiaxin Duan, Hui Jian, Ting Liu, Guanglu Che

**Affiliations:** Department of Laboratory Medicine, West China Second University Hospital, and Key Laboratory of Obstetric & Gynecologic and Pediatric Diseases and Birth Defects of Ministry of Education, Sichuan University, No. 20, Section 3, Renmin South Road, Chengdu, 610041 Sichuan People’s Republic of China

**Keywords:** Enterovirus, HFMD, Epidemiology, Clinical characteristics, Change

## Abstract

**Objectives:**

This study aims to investigate molecular epidemiology and clinical characteristics of enterovirus associated hand-foot-mouth disease (HFMD) in Chengdu, China, 2013–2022. Monitoring the molecular epidemiology and clinical features of HFMD for up to 10 years may provide some ideas for future protection and control measures.

**Methods:**

We conducted a retrospective analysis of the medical records of all patients with laboratory-confirmed HFMD-related enterovirus infection at the West China Second University Hospital from January 2013 to December 2022. We described the characteristics in serotype, age, sex distribution and hospitalization of enterovirus infection cases using data analysis and graphic description.

**Results:**

A total of 29,861 laboratory-confirmed cases of HFMD-related enterovirus infection were reported from 2013 to 2022. There was a significant reduction in the number and proportion of EV-A71 cases after 2016, from 1713 cases (13.60%) in 2013–2015 to 150 cases (1.83%) in 2017–2019. During the COVID-19 pandemic, EV-A71 cases even disappeared. The proportion of CV-A16 cases decreased from 13.96% in 2013–2015 to 10.84% in 2017–2019 and then to 4.54% in 2020–2022. Other (non-EV-A71 and non-CV-A16) serotypes accounted for 95.45% during 2020–2022, with CV-A6 accounting for 50.39% and CV-A10 accounting for 10.81%. Thus, CV-A6 and CV-A10 became the main prevalent serotypes. Furthermore, There was no significant difference in the enterovirus prevalence rate between males and females. The hospitalization rate of EV-A71 patients was higher that of other serotypes. In general, the proportion of HFMD hospitalizations caused by other pathogens except for EV-A71, CV-A16, CV-A10 and CV-A16 was second only to that caused by EV-A71. The proportion of children over 4 years old infected with enterovirus increased.

**Conclusion:**

The incidence of HFMD associated with enterovirus infection has decreased significantly and CV-A6 has been the main pathogen of HFMD in Chengdu area in recent years. The potential for additional hospitalizations for other untested enterovirus serotypes suggested that attention should also be paid to the harms of infections with unknown enterovirus serotypes. Children with HFMD were older. The development of new diagnostic reagents and vaccines may play an important role in the prevention and control of enterovirus infection.

**Supplementary Information:**

The online version contains supplementary material available at 10.1186/s12985-023-02169-x.

## Introduction

Hand-foot-and-mouth disease (HFMD) is a highly contagious viral infection that is primarily characterized by fever, rash on the hands and feet, and blisters in the mouth. The disease is caused by a variety of enteroviruses (EVs), which are mainly transmitted through the fecal–oral route, respiratory tract, or contact with cutaneous lesions [1]. HFMD primarily affects children under the age of 5 and has a higher incidence during the summer and autumn months in temperate regions. In tropical regions, the disease is prevalent year-round [2]. HFMD has a high prevalence worldwide, especially in the Asia–Pacific region [3]. While most cases of HFMD are mild and self-limiting, some cases can progress rapidly and result in severe central nervous system (CNS) complications. In such cases, the disease can deteriorate dramatically, leading to cardiopulmonary failure and even death [4]. For instance, enterovirus 71 (EV-A71) can cause serious neuropathology and cardiopulmonary complications, such as aseptic meningitis, acute flaccid paralysis, brainstem encephalitis, fatal myocarditis, and pulmonary edema, which can ultimately lead to death [5]. Vaccination to prevent Enterovirus 71 infection is key to reducing severe cases and deaths from HFMD, and easing the burden of the disease [6]. In recent years, several inactivated and live attenuated EV-A71 vaccines have been developed and licensed in China. These vaccines have been shown to be safe and effective in clinical trials, and have been implemented in national immunization programs to control and prevent HFMD outbreaks. The inactivated EV-A71 vaccine has been demonstrated to be effective in reducing the incidence of EV-A71-associated diseases, including severe neurological complications, in young children [7]. The domestic EV-A71 vaccine was launched in Chengdu in August 2016. The more data after the introduction of the EV-A71 vaccination should be showed in Chengdu area to help HFMD prevention and control.

HFMD is caused by more than 20 types of EVs, which are a genus of positive-sense single-straned RNA viruses in the family Picornaviridae [8]. Of all HFMD-associated enteroviruses, 90% belong to coxsackievirus (CV) group A. Among them, EV-A71 and coxsackievirus A16 (CV-A16) are the two main serotypes responsible for HFMD [9]. Over the past decade, the majority of EV71-associated HFMD outbreaks occurred in China among the Asia–Pacific countries [10]. Recent epidemic analysis has shown that CV-A6 has replaced CV-A16 and EV-A71 as the leading epidemic serotypes in many provinces of China [11]. This demonstrates that CV-A6 is a major cause of HFMD in patients after the launch of EV-A71 vaccination in Chengdu [12]. However, there is currently a lack of systematic data to simultaneously evaluate the molecular epidemiology and clinical characteristics of HFMD in Chengdu after the EV71 vaccination and the COVID-19 pandemic.

In conclusion, this study provides a comprehensive analysis of the molecular epidemiology and clinical characteristics of HFMD-associated enterovirus infections in Chengdu from 2013 to 2022. Our findings revealed significant changes in the viral spectrum and clinical features during two crucial periods—the introduction of the EV-A71 vaccine and the COVID-19 pandemic. Specifically, the incidence of EV-A71 almost disappeared, and the number and proportion of CV-A16 cases decreased in recent years. Furthermore, the total number of enterovirus infections declined significantly, and CV-A6 emerged as the leading pathogen, surpassing both CV-A10 and CV-A16. The hospitalization rate of EV-A71 patients was higher that of other serotypes. In general, the proportion of HFMD hospitalizations caused by other pathogens except for EV-A71, CV-A16, CV-A10 and CV-A16 was second only to that caused by EV-A71. These findings provide essential information for monitoring the HFMD-associated enterovirus spectrum and epidemiological trends for the development of effective vaccines against EV-A71, CV-A16, CV-A6, CV-A10 and unknown enterovirus serotypes, which maybe more harmful.

## Methods

### Study subjects

We conducted a retrospective analysis of patient records of all cases with laboratory-confirmed Enterovirus Infections (EI) at West China Second University Hospital from 2013 to 2022. The etiological data including age, gender, diagnosis and hospitalization were collected. The study protocol was approved by the Clinical Research Ethics Committee of West China Second University Hospital, Sichuan University. Due to the nature of retrospective studies, informed consent from individual patients is waived.

### Case definition

The diagnosis of HFMD was established based on current epidemiology, clinical manifestation and virological investigation in accordance with the Chinese guidelines for the diagnosis and treatment of HFMD (2018 edition) [13]. In this study, we included all suspected and combined cases of HFMD, including those diagnosed with herpetic angina and other highly suspected or laboratory-confirmed EV infections, due to the overlapping clinical symptoms and similar etiology to HFMD. All laboratory-confirmed EV-positive cases were included in the analyses of this study, except for the positive rate of enterovirus, which was calculated as the number of laboratory-confirmed EV-positive cases in a specific time period compared to the number of possible cases of HFMD in that time period. For the sake of consistency and ease of interpretation, all collected laboratory-confirmed EV-positive cases were uniformly considered as HFMD cases.

### Specimen collection

Specimens, including stool, anal swab and throat swab, were collected from patients and immediately sent to the laboratory in sterile sampling tubes at 0–4 °C. These specimens were stored for no more than 24 h at – 20 °C before testing.

### Laboratory detection

Enterovirus RNA was extracted using the Virus RNA Extraction Reagent (DAAN GENE, China) following the manufacturer’s instruction. The ABI Prism 7500 real-time fuorescence quantitative polymerase chain reaction system (ThermoFisher, USA) was utilized for detecting and analyzing universal enteroviruses including all unclassified serotypes that cause HFMD, EV-A71, CV-A16, CV-A6 and CV-A10. Sansure nucleic acid test kits (Sansure Biotech, China) were used to detect enterovirus universal types, EV-A71, and CV-A16 from 2013 to 2022. From June 1st, 2019 to November 31st, 2022, DAAN nucleic acid test kits (DAAN GENE, China) were employed to detect CV-A6 and CV-A10. The specimen results were determined to be positive or negative following the instructions of the respective kits.

### Hospitalization

We gathered hospitalization data on laboratory-confirmed positive patients with EV infection. Patients exhibiting symptoms such as persistent high fever, impaired mental state, drowsiness, weak suction, hyperexcitability, headache, vomiting, irritability, limb tremors, muscle weakness, and neck stiffness were admitted to the hospital for treatment.

### Statistical analysis

We included all cases in the analysis and stratified them by age group: 0–11 months, 12–23 months, 24–35 months, 36–47 months, 48–59 months, 60–71 months, 72–83 months, 84 months and older. We also stratified the data by enterovirus serotype. To analyze changes in the epidemiology and clinical characteristics of HFMD, we stratified the data by two stages: before versus after the introduction of the EV-A71 vaccine in 2016 and before versus after the COVID-19 pandemic in 2020.

The epidemiological and clinical characteristics of the HFMD were analyzed using descriptive methods. The correlation between the monthly number of cases and the monthly enterovirus positive rate was evaluated using the Pearson rank test. The differences in epidemiological and clinical characteristics of the HFMD between 2013–2015 and 2017–2019 or 2017–2019 and 2020–2022 were assessed using the nonparametric χ^2^ test or Mann Whitney U test. A value of *P* < 0.05 was considered significant. Statistical analyses were performed using GraphPad Prism 8.0 and SPSS software 19.0, and graphical representations were generated using GraphPad Prism 8.0.

## Results

### Overall distribution of enteroviruses in 2013–2022

**Fig. 1:**
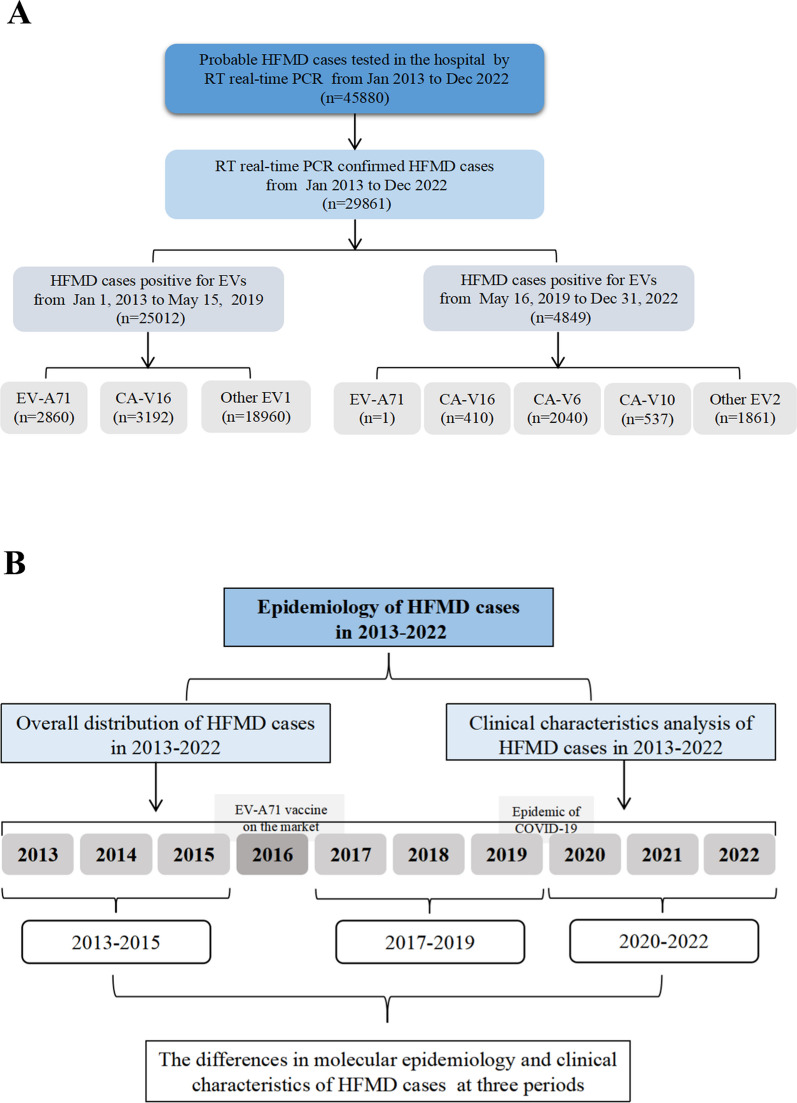
Flowchart about the study. **A** Flowchart illustrating the enrolled HFMD cases during the study period from 2013 to 2022. **B** Flowchart illustrating the several directions of research

**Fig. 2:**
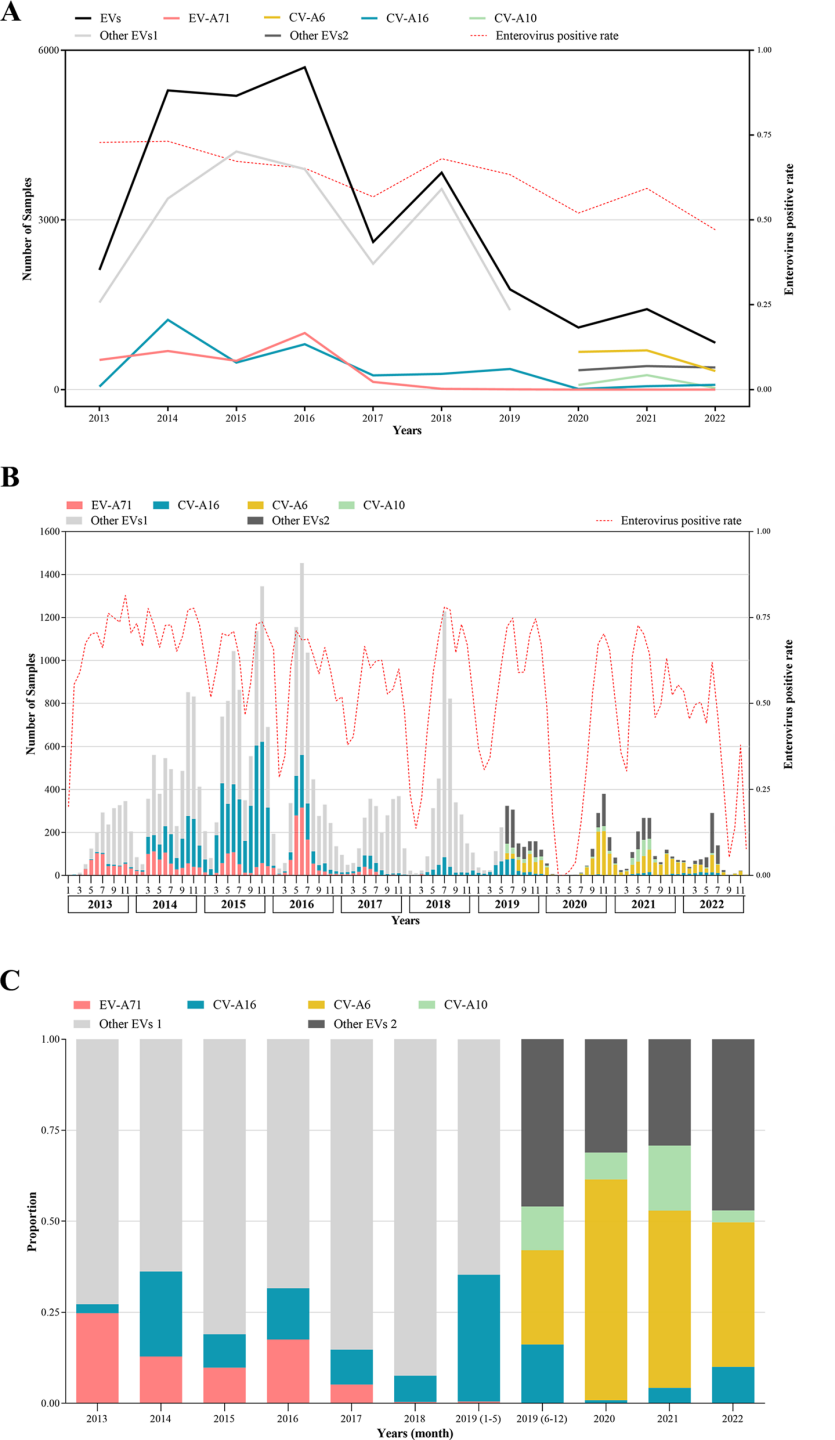
Overall distribution of enteroviruses in 2013–2022. **A** The yearly distribution of laboratory-confirmed HFMD cases in 2013–2022. **B** The monthly distribution of laboratory-confirmed HFMD cases in 2013–2022. **C** The yearly proportion of several common serotypes in laboratory-confirmed HFMD cases in 2013–2022. EVs: All enteroviruses caused HFMD; EV-A71: Enterovirus 71; CV-A16: Coxsackievirus A16; CV-A6: Coxsackievirus A6; CV-A10: Coxsackievirus A10; Other EVs 1: Not including EV-A71 and CV-A16; Other EVs 2: Not including EV-A71, CV-A16, CV-A6 and CV-A10; Enterovirus positive rate: The number of laboratory-confirmed EV-positive cases compared to the number of possible HFMD cases

To study the prevalence of enteroviruses in Chengdu, we conducted a serial of analyses on laboratory-confirmed enterovirus infections caused by CV-A16, EV-A71, CV-A6, CV-A10 and other EVs during 2013–2022. In total, 29,861 laboratory-confirmed cases of enterovirus infection were reported from 2013 to 2022 (Fig. 1A). As shown in the statistical plot of enterovirus infections per year (Fig. 2A), the annual total number of HFMD cases showed a trend of initially increasing and then decreasing, reaching its peak in 2016 and its lowest point in 2022. Following the introduction of the EV-A71 vaccine in 2016, the total number of enterovirus infections in 2017 was half of that in 2016. The changes in the numbers of EV71 and other infections were consistent with the total number of enterovirus infections. Although the number of CV-A16 infections also showed an overall downward trend, the lowest infection level was observed in 2020. The annual positive rate of enterovirus infection from 2013 to 2019 was significantly higher than that from 2020 to 2022.

Further analysis of monthly enterovirus infection characteristics between 2013 and 2022 revealed that HFMD-associated enterovirus infections occurred throughout the year except for March and April 2020, due to the impact of COVID-19 (Fig. 2B). Generally, enterovirus infections were seasonal, with two main infection peaks observed during 2013–2017, 2019, and 2021: one from April to July or August and another from September to December. However, only one peak of infection was observed in 2018, from May to October. Due to the impact of COVID-19, there was almost no enterovirus infection from February to June 2020, with infections peaking in October and November. In 2022, the peak of infection occurred in June and July, and few cases appeared in September and December. The monthly positive rate of enterovirus infection corresponded to the infection peak, with a high positive rate at the peak of infection and a low positive rate during the low peak of infection.

Analysis of the proportion of serotypes of enterovirus infections revealed that the percentage of EV-A71 infections significantly decreased after 2017, and this infection was not observed between 2020 and 2022. The infection rate of CV-A16 was the lowest in 2020, at 0.82%. However, following the addition of CV-A6 and CV-A10 tests in our laboratory in 2019, it was discovered that the infection rate of CV-A6 in 2020 was as high as 60.66%, and the infection rate remained elevated between 2021 and 2022 (Fig. 2C).

### Gender, age and hospitalization characteristics of patients with different serotypes of enterovirus infection

**Fig. 3:**
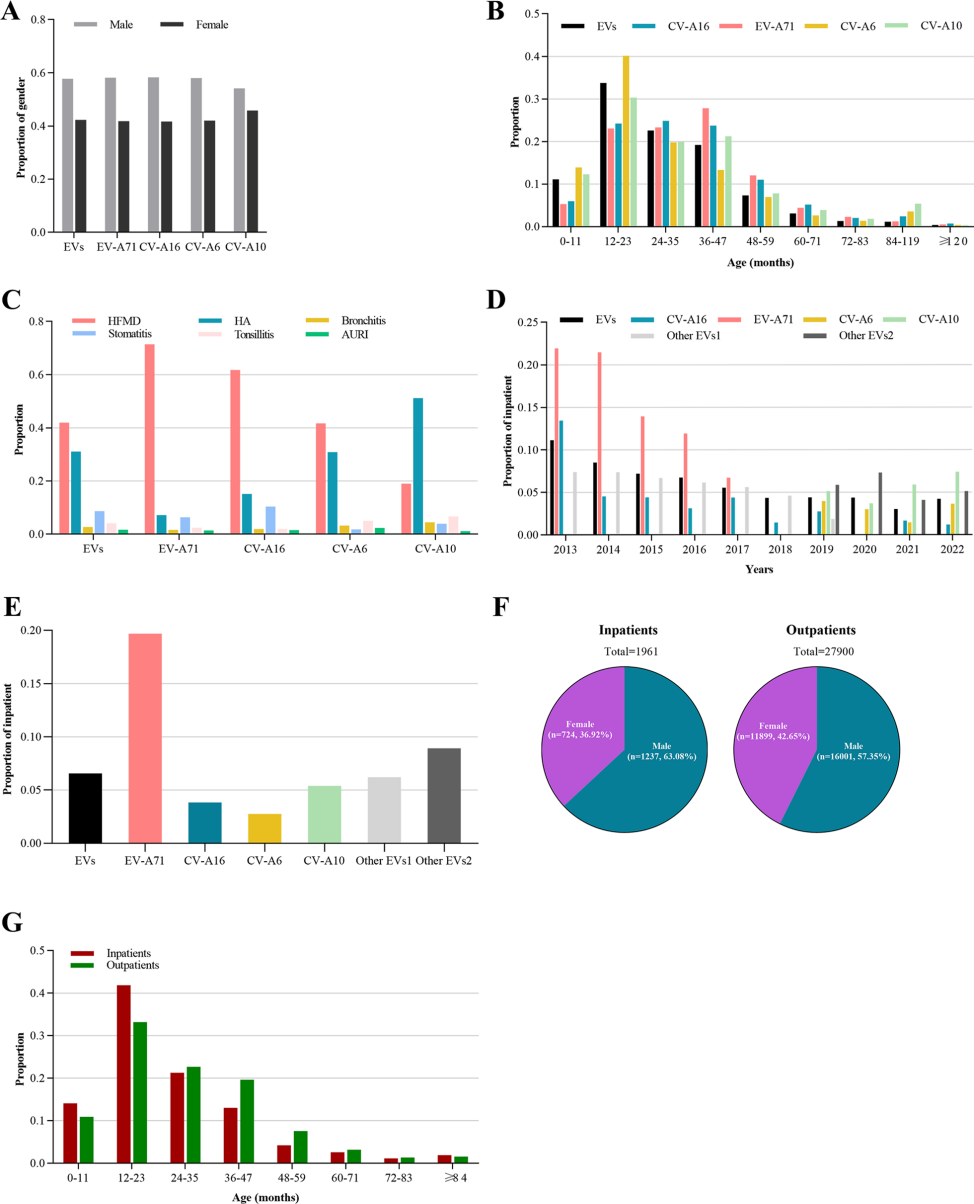
Clinical characteristics of HFMD cases with several common serotypes in 2013–2022. **A** The sex distribution in cases of EVs, EV-A71, CV-A16, CV-A6, and CV-A10, respectively. **B** The age distribution in cases of EVs, EV-A71, CV-A16, CV-A6, and CV-A10, respectively. **C** The main diagnosis in cases of EVs, EV-A71, CV-A16, CV-A6, and CV-A10, respectively. **D** The hospitalization rate in cases of EVs, EV-A71, CV-A16, CV-A6, and CV-A10 each year from 2013 to 2022, respectively. **E** Overall hospitalization rates for EVs, EV-A71, CV-A16, CV-A6, and CV-A10 in 2013–2022. **F** Male to female ratio of HFMD inpatients and outpatients respectively, 2013–2022. **G** The age distribution of HFMD inpatients and outpatients respectively, 2013–2022

We conducted an analysis to gain insight into the clinical characteristics of patients infected with EV-A71, CV-A16, CV-A6, and CV-A10. Our results revealed that there was no significant difference in the proportion of males and females infected with different serotypes and there were more male cases than female cases among population infected with different serotypes (Fig. 3A). Children aged 12–47 months were found to be the most susceptible to enterovirus infections, especially those aged 12–23 months. EV-A71 infected patients were mainly between 36 and 47 months of age, while the age range of CV-A16 infected patients was distributed almost equally among the 12–23, 24–35, and 36–47 months age groups. CV-A6 infected patients were mostly 12–23 months old, accounting for 40.15% of CV-A6 cases, whereas CV-A10 infected patients were also predominantly 12–23 months old, accounting for 30.35% of CV-A10 cases (Fig. 3B). The primary symptoms of both EV-A71 and CV-A16 were HFMD, accounting for 71.44% and 61.72% of cases, respectively. CV-A6 predominantly caused HFMD or herpes angina, accounting for 41.72% and 30.88% of cases, respectively. In contrast, CV-A10 was primarily associated with herpetic angina, accounting for 51.21% of cases (Fig. 3C). Overall, we found that different serotypes of enterovirus in Chengdu displayed variation in the age and symptoms of infected individuals, but showed little variation in the gender of infected individuals throughout the 2013–2022 period.

We conducted a thorough analysis of the percentage of hospitalized patients who were infected with various serotypes of enterovirus between 2013 and 2022 (Fig. 3D–E). Compared with other serotypes, the hospitalization rate of EV-A71 infection was the highest, reaching 21.94% in 2013, and then decreased year by year. Overall, patients infected with CA-V10 had significantly higher hospitalization rates than those infected with CA-V6, while patients infected with all subtypes except EV-A71, CV-A16, CV-A10, and CV-A16 had significantly higher hospitalization rates than those infected with CA-V10. The proportion of male inpatients was slightly higher than that of non-inpatients (Fig. 3F). The proportion of hospitalized patients aged 0–23 months was slightly higher than that of non-hospitalized patients (Fig. 3G).

In addition, comparing the clinical features of enterovirus positive and negative cases found that the two groups of cases of gender, age, hospitalization rate and major clinical symptoms had significant difference (Additional file 1: Table S1). Compared with enterovirus negative cases, positive cases had more male ratio and lower hospitalization rate. The age of positive cases was more concentrated between 12 and 47 months old, and the main clinical diagnosis of more than 73% of positive cases was HFMD or herpangina.

### The changes in molecular epidemiology and clinical characteristics of hand-foot-mouth disease associated enterovirus infection in Chengdu at different periods

The period between 2013 and 2022 marked two significant events, the introduction of the EV-A71 vaccine in 2016 and the outbreak of the novel coronavirus towards the end of 2019. To provide a comparative analysis of the epidemiological and clinical characteristics of enterovirus infection, we divided the period into three sub-periods: 2013–2015, 2017–2019, and 2020–2022 (Fig. 1B). The results of our analysis are presented in Table 1.

**Table 1:** Characteristic changes of HFMD cases in 2013–2015, 2017–2019 and 2020–2022 of chengdu area

Characteristics	2013–2015 (N = 12,599)	2017–2019 (N = 8216)	2020–2022 (N = 3348)	*P*1 value	*P*2 value
Gender, n(%)				0.019^a^	0.334^a^
Male	7317 (58.08)	4636 (56.43)	1922 (57.41)		
Female	5282 (41.92)	3580 (43.57)	1426 (42.59)		
Male Female	1.39	1.29	1.34		
Median age, month (P25, P75)	26 (16–36)	25 (15–36)	25 (16–36)	0.331^b^	< 0.001^b^
Age group,month, n (%)				< 0.001^a^	< 0.001^a^
0–11	1335 (10.60)	1069 (13.01)	372 (11.11)		
12–23	4329 (34.36)	2868 (34.91)	1170 (34.95)		
24–47	5658 (44.91)	3105 (37.79)	1201 (35.87)		
> 48	1277 (10.14)	1174 (14.29)	605 (18.07)		
Serotypes, n (%)				< 0.001^a^	< 0.001^a^
EV-A71	1713 (13.60)	150 (1.83)	0 (0)		
CV.A16	1759 (13.96)	891 (10.84)	152 (4.54)		
Other enterovirus 1	9127 (72.44)	7175 (87.33)	3196 (95.45)		
CV-A6	–	–	1687 (50.39)		
CV-A10	–	–	362 (10.81)		
Other enterovirus 2	–	–	1147 (34.26)		
In/outpatient, n (%)				< 0.001^a^	0.020^a^
Inpatient	1061 (8.42)	390 (6.73)	126 (3.76)		
Outpatient	11,538 (91.58)	7826 (93.27)	3222 (96.24)		

Other enterovirus l: No-EV-A71 andNo-CV-A16 enterovirus; Other enterovirus 2: No-EV-A71_;_ No-CV-A16_;_ N0-CV-A6 and No-CV-AlO entennirus; *P*1 value: the data in 2017–2019 compared with 2013–2015; *P*2 value: the data in 2020–2022 compared with 2017–2019; ^a^means Chi-square test was used for statistical analysis; ^b^means Mann Whitney U test was used for statistical analysis:"–" indicates that no data of CV-A6 and CV-A10 were collected during 2013–2019

The total number of enterovirus cases demonstrated a significant decrease during the three analyzed periods: 2013–2015, 2017–2019, and 2020–2022. Specifically, the numbers and proportions of EV-A71 cases followed a consistent downward trend across all three periods, and the virus even disappeared at 2020–2022. Similarly, the numbers and proportions of CV-A16 cases decreased between 2013–2015 and 2017–2019, and then showed a further significant reduction in 2020–2022. Consequently, the proportion of other enterovirus cases increased to 95.45% at 2020–2022, with CV-A6 cases accounting for 50.39% of them. In conclusion, the prevalence of CV-A16 and EV-A71 cases decreased dramatically, and CV-A6 became the dominant serotype during the 2020–2022 period.

After the introduction of the EV-A71 vaccine and the outbreak of the novel coronavirus at the end of 2019, the age of HFMD patients tended to be older. The sex ratio of enterovirus-infected people remained consistent across the three periods. Our analysis revealed that the total age distribution differed between the three periods. In 2017–2019, the proportion of patients aged 0–11 months and over 48 months increased compared to 2013–2015, while the proportion of patients aged 24–47 months decreased. In 2020–2022, the proportion of patients aged 0–11 months decreased compared to 2017–2019, while the proportions of patients aged over 48 months increased.

The age changes of patients with different serotypes of infection were further analyzed (Table 2). For patients infected by EV-A71 and CV-A16, the proportion of patients aged over 48 months increased significantly in 2017–2019 compared with 2013–2015, while there were reduced in the age group of 12–47 months. During 2020–2022 compared with 2017–2019, no significant age changes were found in CV-A16 patients, except for patients 24–47 months and over 48 months old. For patients infected by other EV1s, only the proportion of patients aged 24–47 months decreased significantly during 2017–2019 compared with 2013–2015, while there was a significant increase in the proportion of patients aged 0–11 months and over 48 months. Compared with 2017–2019, the proportion of other EV1s patients under 1 year old dropped dramatically during 2020–2022. Meanwhile, the proportion of other EV1s patients over 48 months old continued to increase during 2020–2022 compared with 2017–2019.

**Table 2:** The age distribution and hospitalization with different serotypes in 2013–2015, 2017–2019 and 2020–2022 of Chengdu area

	2013–2015	2017–2019	2020–2022	*P*1 value	*P*2 value
EV-A71.N					
Age group, month n (%)				< 0.001	–
0–11	99 (5.78)	12 (8.00)	–		
12–23	434 (25.34)	27 (18.00)	–		
24–47	893 (52.13)	58 (38.67)	–		
> 48	287 (16.75)	53 (35.33)	–		
In outpatient, n (%)					
Inpatient	332 (19.38)	9 (6.00)	–		
Outpatient	1381 (80.62)	141 (94.00)	–		
CV-A16.N	1759	891	152		
Age group,month n (%)				< 0.001	0.225
0–11	106 (6.03)	56 (6.29)	7 (4.61)		
12–23	457 (25.98)	193 (21.66)	31 (20.39)		
24–47	942 (53.55)	369 (41.41)	47 (30.92)		
≥ 48	254 (14.44)	273 (3064)	67 (44.08)		
In outpatient, n(%)				0.016	0.285
Inpatient	84 (4.78)	25 (2.81)	2 (1.32)		
Outpatient	1675 (95.22)	866 (97.19)	150 (98.68)		
Other enterovirus 1, N	9127	7175	3196		
Age group, month n (%)				< 0.001	< 0.001
0–11	1130 (12.38)	1001 (13.95)	365 (11.42)		
12–23	3438 (37.67)	2648 (36.91)			
24–47	3823 (41.89)	2678 (37.32)			
> 48	736 (8.06)	848 (11.82)			
In outpatient, n(%)				< 0.001	0.016
Inpatient	645 (7.07)	356 (4.96)	124 (3.88)		
Outpatient	8482 (92.93)	6819 (95.04)	3072 (96.12)		

Other enterovirus 1: No-EV-A71 and No-CY-A16 enterovirus; *P*1 value: the data in 2017–2019 compared with 2013–2015; *P*2 value: the data in 2020–2022 compared with 2017–2019; The chi-square test was used for all analysis in the Table 2

Compared to the period between 2013 and 2015, the hospitalization rate of patients with laboratory-confirmed enterovirus-positive infections continued to decline from 2017–2019 to 2020–2022 (Table 1). The hospitalization rate of patients with EV-A71 infection decreased significantly from 19.38% during the period from 2013 to 2015 to 6.00% during the period from 2017 to 2019. Similarly, from 2013–2015 to 2017–2019, the hospitalization rates of patients infected with CA-V16 and other serotypes also decreased. The hospitalization rate of patients infected with other serotypes decreased significantly from 2017–2019 to 2020–2022 (Table 2).

## Discussion

This study described the molecular epidemiology and clinical features of HDMD-associated enterovirus in the Chengdu region, 2013–2022. Although the data in this study was limited to one hospital, a pediatric medical center in southwestern China, its medical data has been representative of Chengdu. The analysis was based on cases confirmed positive for enterovirus infection in the laboratory. There may be some deviation in analyzing the incidence of HFMD in Chengdu, but the number of cases confirmed positive for enterovirus infection in this study was greater than in Tang's previous research [14], which covered the city's data. Therefore, the analysis about the molecular epidemiology and clinical characteristics of HFMD in this study was relatively reliable. The study presents the monthly distribution of lab-confirmed HFMD cases along with the patients' age, gender, diagnostic information and hospitalization from 2013 to 2022. Notably, the total number of HFMD cases reached its highest level in 2016, possibly due to the newly introduced EV71 vaccine, which had not yet been widely adopted or had not had a chance to take effect. Consequently, the HFMD-related enterovirus continued to spread extensively in 2016, leading to a sharp decrease in HFMD cases in 2017 due to the vaccine's efficacy or increased attention for HFMD. Subsequently, the number of HFMD cases rose sharply in 2018, followed by a continuous decline until 2022. These trends are consistent with those reported in other studies [13, 15, 16]. It is noteworthy that the number of HFMD cases remained low from 2020 to 2022, particularly from February to June 2020 and from September to December 2022, mainly due to the strict control measures against COVID-19, which has reduced the chances of children coming into contact with infected or potentially infected children. Similar results were found in Song’s study [17]. This finding provides valuable evidence for future prevention and control of HFMD-related enterovirus infections. It should be noted that studies based on other articles have shown that total hospital admissions, particularly for less severe illnesses, have decreased during COVID-19 [18, 19]. This may result in a smaller number of HFMD cases being reported during the pandemic. Interestingly, we observed a correlation between the number of HFMD cases per month and the positive rate of enterovirus infection in that month. This indicates that the diagnosis was more effective during periods of high HFMD incidence, highlighting the need to enhance diagnostic capabilities during periods of low incidence.

Studies have reported that the EV-A71 vaccine plays a crucial role in reducing the number of HFMD cases and the severity rate and mortality rate caused by EV-A71 [7, 8, 20–22]. Consistent with previous observations, our study found a significant decrease in the number, proportion and hospitalization rate of HFMD cases infected with EV-A71 after the launch of the EV-A71 vaccine, with the virus even disappearing during the COVID-19 pandemic. It should be pointed out that not only the number of HFMD cases caused by EV-A71 has decreased, but also the total number of HFMD cases, indicating that in addition to the role of vaccines, other factors such as the improvement of people's health awareness and the reduction of the spread of HFMD have also played an important role in reducing HFMD. Moreover, the number and proportion of cases caused by CV-A16 also significantly reduced following the EV-A71 vaccination and during COVID-19 outbreaks. However, the proportion of HFMD cases caused by other EVs increased after the EV-A71 vaccination and during COVID-19 outbreaks. Subsequently, after the detections of CV-A6 and CV-A10 were added in June 2019, we found that CV-A6 cases accounted for more than half of the total cases during COVID-19, making it the dominant serotype. Similarly, HFMD outbreaks by CV-A6 have been reported worldwide in recent years, including in China [23]. While we cannot determine whether CV-A6 was the dominant pathogen in the Chengdu area before June 2019 due to the absence of a CV-A6 test, available data indirectly suggested that the development of a multivalent vaccine and appropriate public health interventions could help prevent and control enterovirus infection more effectively. A main limitation of this study was the lack of analysis on the vaccination status of patients with HFMD against EV-A71, which prevented direct confirmation of the effectiveness of the EV-A71 vaccine in preventing and controlling HFMD. However, based on other researches about the effect of the EV-A71 vaccine [14, 24], we can indirectly draw the conclusion from this study that the infection and hospitalization of EV-A71 cases have decreased after 2016 in the Chengdu area. In future study, we hope that we can investigate how the molecular changes of EV-A71 virus after the available of its vaccine in 2016 when compared with without vaccine. Another limitation was that the study was a descriptive analysis, and we cannot explain the mechanism of epidemiological changes, such as why the number and proportion of CV-A16 cases have also decreased, which may be related to the mutation avoidance mechanism of the virus itself. Of course, we hope that there will be opportunities in subsequent studies to figure out the reasons for the changes. Therefore, we believe that the use of the EV-A71 vaccine and control measures of COVID-19 have to some extent influenced the changes in the enterovirus profile of HFMD. It also needs to be discussed that hospitalization rates of HFMD patients with CV-A16 and other EV1s were also reduced during 2017–2019 and during 2020–2022. Possible reasons for this include the people's timely medical treatment and the improvement of medical technology, resulting in a decline in overall hospitalization rates. On the other hand, we also found hospitalization rates with other EV2s infection was second high than that with EV-A71infection. So the more attention should be paid to other EV2s infection, which not include EV-A71, CV-A16, CV-A10 and CV-A16.

To further investigate the clinical characteristics of HFMD, we analyzed the gender, age, and disease diagnosis or signs of patients infected with different serotypes. Our analysis found that the gender of HFMD patients was not significantly affected by infection serotype, EV71 vaccination [25] or COVID-19. However, the main diagnosis of enterovirus infection associated with HFMD varied among serotypes. HFMD was mainly diagnosed after enterovirus infection, represented by EV-A71 and CV-A16, while herpes angina was mainly diagnosed after CV-A10 infection. According to our age analysis of laboratory-confirmed positive patients from 2013 to 2022, the age of HFMD patients was mainly under 5 years old, with the largest number between 12 and 23 months, followed by 24–47 months and 0–11 months. The age distribution of different serotypes was different, with the proportion of CV-A6 and CV-A10 patients under 2 years old being higher than that of EV-A71 and CV-A16 patients. This suggests that the age of CV-A6 and CV-A10 patients is younger than that of EV-A71 and CV-A16 patients. Additionally, we found the great difference in the clinical features between enterovirus positive and negative cases. From the fact that the main symptoms of EV-negative cases were not HFMD or herpangina, it can be inferred that the majority of pathogens in EV-negative cases were not enterovirus.

During the period from 2017 to 2019, we found that the proportion of patients decreased significantly only between 24 and 35 months of age, while the proportion of patients younger than 1 year of age and 48 months and older increased significantly. This is different from several studies that reported a reduction in patients under 1 year of age after the launch of EV-A71 vaccine [25–27]. As our previous analysis found differences in age distribution among different serotypes, we speculated that the increased proportion of patients under 1 year old after EV-A71 vaccination might be due to the influence of other serotypes except EV-A71 on the age of infected patients. Indeed, our analysis revealed a significant increase in the proportion of patients under 1 year of age with other enterovirus infections (non-EV-A71 and non-CV-A16) during 2017–2019. However, we also found that compared to 2013–2015 when EV-A71 vaccine was not yet available, the proportion of EV71 patients under 1 year old increased between 2017 and 2019, possibly due to the small number of EV71 patients infected during that period, leading to statistical error. Additionally, the reason for the data deviation may be that patients in this age group were more likely to be referred to superior hospitals like ours for diagnosis and treatment.

## Conclusion

There was great changes in the number, enterovirus serotypes, hospitalization rate and age of patients with HFMD in 2013–2022. The overall number of HFMD cases showed a decreasing trend, among which EV-A71 infection almost disappeared, instead, CV-A6 infection accounted for more than 50%. The proportion of children over 4 years old infected with enterovirus increased, suggesting that children with HFMD were older. The hospitalization rate of other unknown enterovirus serotype infections was second only to EV-A71, suggesting that we need to pay more attention to other unknown enterovirus serotypes. In the past 10 years, the public's awareness of prevention and control of HFMD has been continuously improved, which may has affected the spread of HFMD. EV-A71 vaccine has also been proved to play an unprecedented positive role in the prevention and control of HFMD. In addition, public prevention and control measures for the novel coronavirus have further suppressed the occurrence of HFMD. Based on these findings, future development of multivalent enterovirus vaccines based on monitoring the pathogen spectrum of HFMD and promoting the prevention of enterovirus infections through personal protective measures are of great importance to reduce the impact of HFMD on our health.

### Supplementary Information

**Additional file 1**. Comparison of clinical characteristics between enterovirus positive and negative cases confirmed by PCR test.

## Data Availability

Not applicable.
