# Supplementary figures and images for: Molecular epidemiology and clinical characteristics of enteroviruses associated HFMD in Chengdu, China, 2013–2022
Source: Virol J. 2023 Sep 3;20:202. doi: 10.1186/s12985-023-02169-x (PMC10476316; doi:10.1186/s12985-023-02169-x)

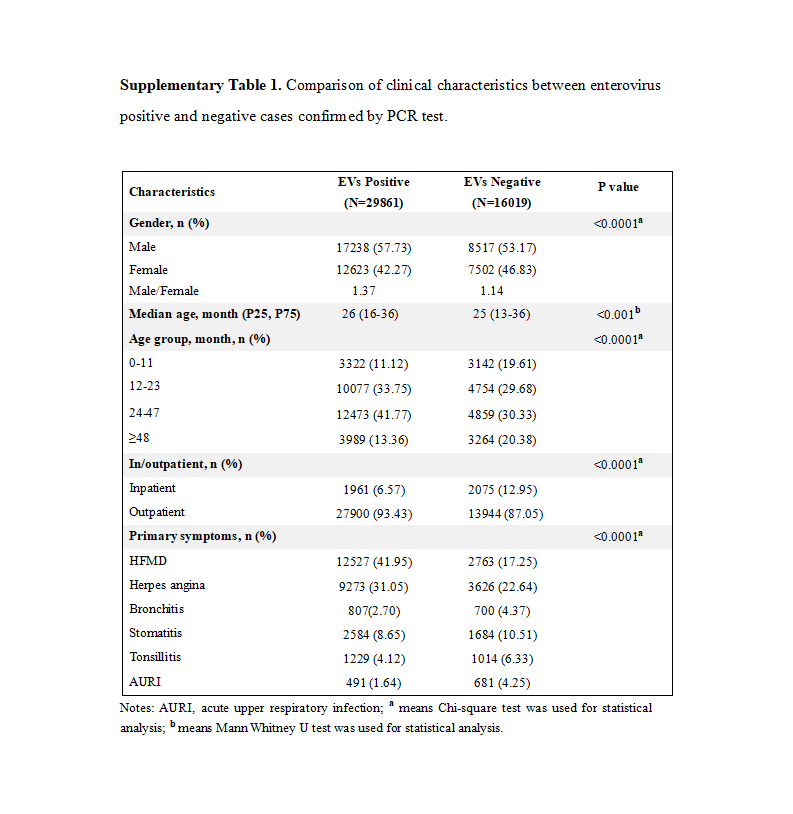

Supplement: Supplementary file 1 — Additional file 1. Comparison of clinical characteristics between enterovirus positive and negative cases confirmed by PCR test. [file 12985_2023_2169_MOESM1_ESM.tif]
